# Fluorescence of the Retinal Chromophore in Microbial and Animal Rhodopsins

**DOI:** 10.3390/ijms242417269

**Published:** 2023-12-08

**Authors:** Dmitrii M. Nikolaev, Andrey A. Shtyrov, Sergey Yu. Vyazmin, Andrey V. Vasin, Maxim S. Panov, Mikhail N. Ryazantsev

**Affiliations:** 1 Institute of Chemistry, Saint Petersburg State University, 26 Universitetskii pr, 198504 St. Petersburg, Russia; 2 Nanotechnology Research and Education Centre RAS, Saint Petersburg Academic University, 8/3 Khlopina Street, 194021 St. Petersburg, Russia; 3 Institute of Biomedical Systems and Biotechnologies, Peter the Great St. Petersburg Polytechnic University, 29 Polytechnicheskaya Str., 195251 St. Petersburg, Russia; 4 Center for Biophysical Studies, St. Petersburg State Chemical Pharmaceutical University, Professor Popov str. 14, lit. A, 197022 St. Petersburg, Russia

**Keywords:** rhodopsins, fluorescent rhodopsins, fluorescence spectroscopy

## Abstract

Fluorescence of the vast majority of natural opsin-based photoactive proteins is extremely low, in accordance with their functions that depend on efficient transduction of absorbed light energy. However, several recently proposed classes of engineered rhodopsins with enhanced fluorescence, along with the discovery of a new natural highly fluorescent rhodopsin, NeoR, opened a way to exploit these transmembrane proteins as fluorescent sensors and draw more attention to studies on this untypical rhodopsin property. Here, we review the available data on the fluorescence of the retinal chromophore in microbial and animal rhodopsins and their photocycle intermediates, as well as different isomers of the protonated retinal Schiff base in various solvents and the gas phase.

## 1. Introduction

**Figure 1:**
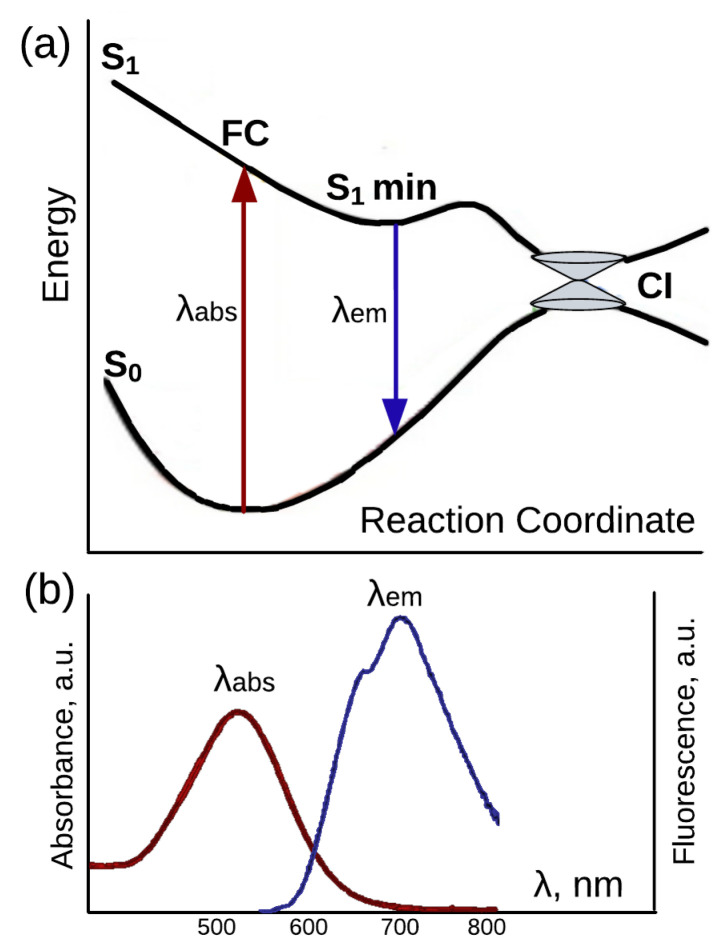
(**a**) Potential energy profiles along the reaction coordinate for the ground state ($S_{0}$) and the excited state ($S_{1}$) of the retinal chromophore for fluorescent rhodopsins. Light absorption promotes retinal from the $S_{0}$ to the $S_{1}$ potential energy surface. From the Franck–Condon (FC) region of $S_{1}$, the excited state relaxes to the minimum on the $S_{1}$ surface, from which it can either return to the $S_{0}$ state through a radiative decay or proceed further to the conical intersection (CI). At the CI, a non-radiative transition to the $S_{0}$ surface occurs, and the reaction path branches leading either to the successful isomerization or back to the initial form of the retinal. (**b**) Absorption and fluorescence spectral bands of proteorhodopsin. The figure was composed by the authors using data from Ref. [13].

Rhodopsins are heptahelical photosensitive membrane-embedded proteins that have been found in organisms from all domains of life [1,2,3,4,5,6]. The rhodopsin functioning cycle starts with light absorption by the retinal cofactor, which is covalently bound to the conserved lysine of apoprotein via the Schiff base linkage. Photon absorption converts the retinal to the first excited state, and the decay to the ground state can proceed either through non-radiative or radiative pathways. The non-radiative decay goes through a conical intersection where the branching of the photoreaction occurs (Figure 1 and its legend). The first path converts the chromophore back to the initial form, and the second path leads to the successful photoisomerization reaction that initiates a series of processes required for a rhodopsin to perform its biological function. In rhodopsins, the protein environment facilitates the photoisomerization of a chromophore, since it is the process that is directly related to rhodopsin functioning, and, as a rule, photoisomerization in rhodopsins occurs very efficiently (e.g., photoisomerization quantum yields in bovine visual rhodopsin and bacteriorhodopsin are 0.65 and 0.64, respectively) [7,8,9]. On the contrary, fluorescence is a side process for rhodopsin functions, and the radiative pathway is suppressed in these proteins. Observed fluorescence quantum yields of microbial and animal rhodopsins are extremely low, with a single exception of the recently discovered neorhodopsin (NeoR) from *Rhizoclosmatium globosum* [10,11,12].

Recent studies on the application of microbial rhodopsins as fluorescent genetically encoded indicators of cell membrane potential facilitated the development of bright rhodopsin variants. A directed evolution approach has allowed researchers to obtain rhodopsin mutants with significantly more intense fluorescence than observed in the wild-type rhodopsins at physiological pH, and engineered proteins have been successfully applied in a large number of biological and medical studies [14,15,16,17,18,19,20,21]. An increasing number of applications of bright rhodopsin variants have raised the interest in rhodopsin fluorescence properties [22,23,24]. In this review, we compile, systematize, and analyze experimental data obtained for fluorescence properties of rhodopsins that are available in the literature. The studies on microbial and animal rhodopsins and their mutants, rhodopsin photocycle intermediates, and also the retinal protonated Schiff base in solvents and the gas phase are considered.

## 2. Fluorescence Properties of Microbial Rhodopsins

**Figure 2:**
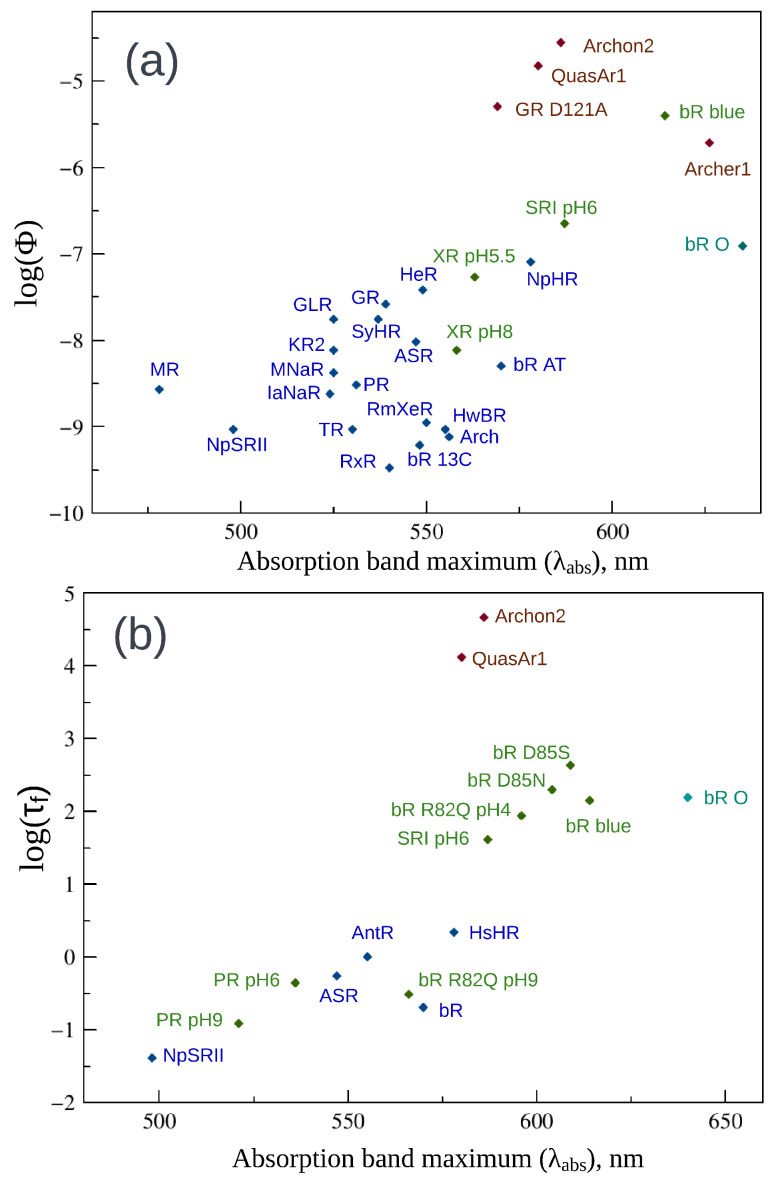
(**a**) Fluorescence quantum yields logarithm (log Φ) of microbial rhodopsins as a function of absorption band maxima ($\lambda_{abs}$). (**b**) Fluorescence lifetimes logarithm (log $\tau_{f}$) of microbial rhodopsins as a function of $\lambda_{abs}$. Wild-type microbial rhodopsins at physiological pH are shown in blue. Microbial rhodopsins at non-physiological pH and rhodopsin mutants except engineered bright mutants are shown in green. The bacteriorhodopsin O photocycle intermediate is shown in light blue. Bright mutants of archaerhodopsin-3 and *Gloebacter violaceus* rhodopsin are shown in brown. For abbreviations of proteins, see Table 1.

Microbial rhodopsins, which include type 1 rhodopsins and recently discovered heliorhodopsins, perform versatile functions in unicellular microorganisms and viruses, acting as light-driven ion pumps, ion channels, enzymes, or sensors. Microbial rhodopsins are also a key tool in optogenetics where these proteins are used for activation, silencing, or monitoring the electrical activity of cells. Regarding the photosensitive cofactor chromophore, most microbial rhodopsins utilize all-*trans*, 15-*anti* retinal. For fluorescence properties of microbial rhodopsins, in contrast to the animal opsin-based photoreceptive proteins discussed in the next section, a substantial amount of data are available. These data are compiled in Table 1 and represented graphically in Figure 2a,b and 5a,b. Table 1 contains the fluorescence excitation maxima ($\lambda_{exc}$), maxima of fluorescence band ($\lambda_{em}$), quantum yields of fluorescence (Φ), and excited state lifetimes ($\tau_{f}$) that are reported for wild-type microbial rhodopsins and their mutants measured in a wide range of pH values, as well as for the O and Q photocycle intermediates of bacteriorhodopsin.

**Figure 3:**
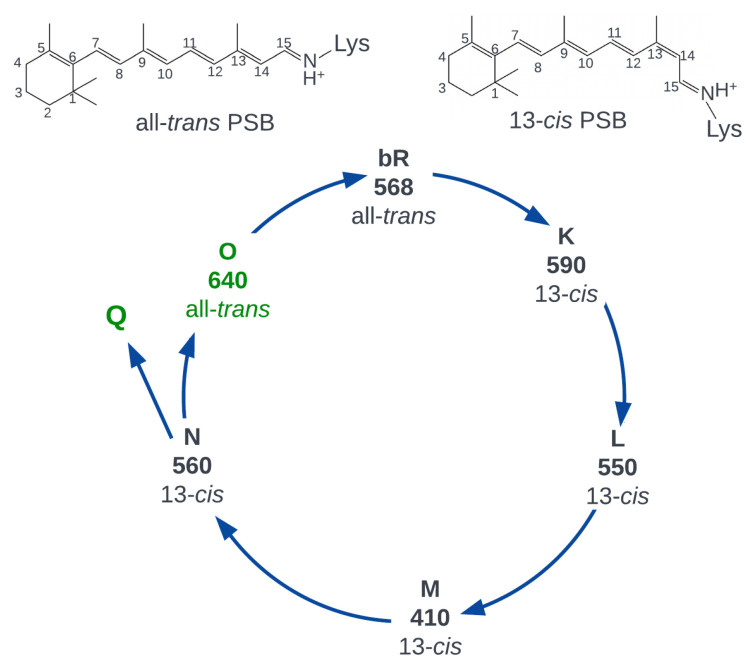
The photocycle of bacteriorhodopsin. After the light-induced photoisomerization reaction described in Figure 1, bacteriorhodopsin returns to the initial state through a series of metastable states called photocycle intermediates. The Q intermediate is not involved in the main photocycle of bacteriorhodopsin and it is generated by photoexcitation of the N intermediate. Numbers denote the absorption maxima of the intermediates. O and Q photocycle intermediates, shown in green, possess enhanced fluorescence.

*Fluorescence quantum yields and fluorescence lifetimes*. Figure 2a shows plots of the fluorescence quantum yields logarithm (log Φ) as a function of corresponding absorption band maxima ($\lambda_{abs}$). The plot is color-coded in the following way. The data for wild-type microbial rhodopsins measured at physiological pH values are represented by blue points. The green color is used to represent data for wild-type microbial rhodopsins measured at pH values other than physiological, as well as for mutants, except the data for highly fluorescent engineered rhodopsins that are shown in brown. Finally, the light blue point in the figures represents the O photocycle intermediate of bacteriorhodopsin (Figure 3). The fluorescence quantum yield of a recently discovered NeoR (Φ=0.2) stands out significantly among the rest of microbial rhodopsins and the corresponding point is omitted in Figure 2a, and the values are given only in Table 1.

Fluorescence quantum yields of discussed rhodopsins vary in a wide range. A tendency of increasing fluorescence quantum yield together with the increasing of the absorption band maximum (but not a strong correlation) can be clearly seen from the plot. This observation suggests that the same factors might be involved in the tuning of these properties. Φ values for the majority of wild-type microbial rhodopsins at physiological pH have the same order of magnitude ∼10${}^{-4}$. The fluorescence quantum yield substantially increases for the acidic forms of bacteriorhodopsin (bR blue, Φ = 4.5 × 10${}^{-3}$) and sensory rhodopsin (SRI pH6, $\Phi =1.3\times 10^{-3}$) reaching the next order of magnitude. The same increase was found for the O and Q photocycle intermediates of bacteriorhodopsin with quantum yields of ≈1 $\times \;10^{-3}$ and $7\times 10^{-3}$, respectively. Because $\lambda_{abs}$ is not measured for the Q intermediate the corresponding point is not shown in Figure 2. Fluorescence quantum yields for three engineered bright variants (Archer1, QuasAr1, and GR D121A) fall in the ∼10${}^{-3}$ region and even achieve the next order of magnitude for Archon2 ($\Phi =1.05\times 10^{-2}$).

A similar picture can be observed for the fluorescence lifetimes of microbial rhodopsins (Table 1). The data are visualized in Figure 2b, where logarithms of fluorescence lifetimes log($\tau_{f}$) are plotted as a function of corresponding absorption band maxima ($\lambda_{abs}$). Again, the points representing the engineered archaerhodopsin-3 mutants (QuasAr1 and Archon2), the O intermediate of bR, two microbial rhodopsins at lower pH (SRI pH6, bR R82Q pH4), and two bR mutants with a substituted counterion (bR D85S, bR D85N) are located above the wild-type rhodopsins at neutral pH and shifted to the right side of the figure. Engineered bright archaerhodopsin-3 mutants QuasAr1 and Archon2 with the largest values of $\tau_{f}$ reported to date are located in the top of the figure.

**Figure 4:**
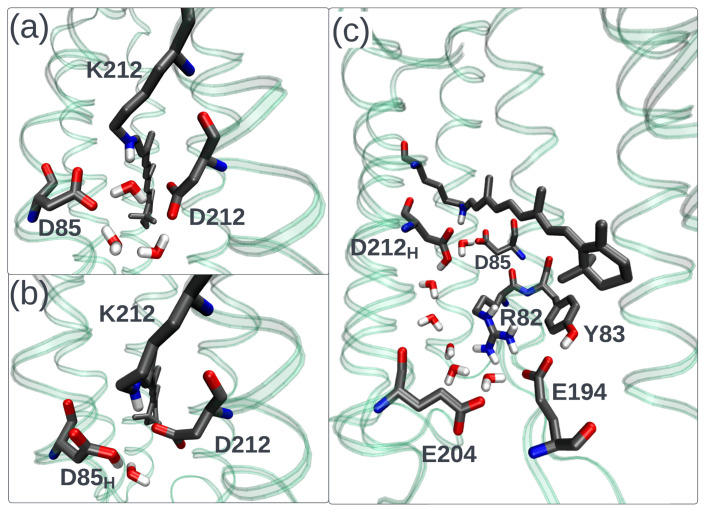
(**a**) The pentagonal cluster composed of two counterions (D85 and D212) and three water molecules in the vicinity of the Schiff base of bacteriorhodopsin at a physiological pH. The structure was taken from Ref. [70]. (**b**) The same region of bacteriorhodopsin at pH ≈ 2 (the blue form of bacteriorhodopsin). (**c**) The structure of bacteriorhodopsin O intermediate. The structure was constructed using homology modeling [71] based on the X-ray structure of O intermediate of bacteriorhodopsin L93A mutant (PDB ID 3VI0).

Clearly, the observed variety of fluorescence properties of opsin-based photoreceptors that contain the same chromophore is determined by difference in the interaction of this chromophore with its environment. The role of such an interaction in tuning rhodopsins’ absorption band maxima is investigated in many studies (e.g., [57,58,59,60,61]). The electrostatic and steric parts of the protein–chromophore interaction are found to be the most important, although in some computational studies [57], non-negligible transfer of electron density between the chromophore and surrounding amino acids was also detected. The most significant color determinant is the electrostatic effect of counterions, the negatively charged amino acids situated in the vicinity of the positively charged ${}^{+}$N-H part of the chromophore, e.g., two titratable residues, D85 and D212, in bacteriorhodopsin (Figure 4a). Both experimental and computational studies demonstrate that a substitution of a counterion by a neutral residue or its protonation leads to a significant red shift of the absorption band maximum. The key role of the counterion electrostatic effect for rhodopsins’ fluorescent properties is also evident from available experimental data. Indeed, the data compiled in Figure 2a,b clearly demonstrates the increase in the fluorescence quantum yields and fluorescence lifetimes with the transition from the ground form of wild-type microbial rhodopsins to the mutants with a counterion substituted by a neutral amino acid or to the form with a neutralized protonated counterion.

Available data on the pH dependence of fluorescent properties also confirm the connection of the counterion protonation state with fluorescence and photoisomerization efficiency. In the study on bacteriorhodopsin [29], a significant increase in fluorescence intensity was detected at acidic pH values with the most intense emission at pH around 1.7 (≈15-fold enhancement compared to neutral pH). Observed pH-dependence of fluorescence intensity correlates with the pH-dependence of the absorption band maximum that shifts to the longer wavelength region upon acidification. On the contrary, only negligible changes in fluorescence intensity and the absorption band maximum were observed in the alkaline pH range. These findings are in line with the pKa value ≈2.7 reported for the bacteriorhodopsin counterion D85 [62]. In a recent time-resolved spectroscopy study on bacteriorhodopsin [30], a slow decay component ($\tau_{f}=7.8$ ps) arising at low pH < 4 has been detected and assigned to the state with a protonated counterion (the blue form, Figure 4b). Similarly, emission intensity enhancement upon lowering the pH value was found for *Gloebacter violaceus* rhodopsin [45], xanthorhodopsin [44], *Exiguobacterium sibiricum* rhodopsin (ESR) [47], and elongation of the excited state lifetime upon acidification was reported for proteorhodopsin (PR) [30,43,63] and Krokinobacter rhodopsin 2 [64].

In all cases, an increase in fluorescence quantum yields or excited state lifetimes occurs in the region close to the pKa of the counterion. Moreover, it was demonstrated that fluorescence intensity can be changed by tuning the counterion pKa [36,47]. For instance, in the study [36], the pKa of the D85 counterion in bacteriorhodopsin was raised up to ≈7.5 by a substitution of the positively charged arginine R82 with a polar glutamine residue. Accordingly, a significant elongation of excited state decay was observed upon lowering the pH value from 9.6 (0.6 ps) to 4.4 (biphasic decay, equally contributing components with lifetimes 2.0 and 7.0 ps). In the study by [47], different magnitudes of fluorescence intensity increase were observed upon lowering the pH value for wild-type ESR and its H57M mutant. In the wild-type protein with the pKa of the D85 counterion estimated to be <2 lowering pH from 7 to 5 resulted in a 2-fold emission enhancement. When a histidine, which strongly interacts with the D85 counterion in ESR, was substituted with methionine, a large increase in pKa (D85) up to 6.3 and, accordingly, an approximately 100-fold increase in fluorescence intensity upon lowering pH from 9 to 4.5 was observed.

An increase in the counterion pKa and, subsequently, enhancement of the fluorescence was also observed for the O intermediate of the bacteriorhodopsin photocycle. The O intermediate arises at the last stage of the photocycle (Figure 3 and converts to the ground state of the protein on the timescale of ≈10 ms [65,66]. The main difference between the O intermediate and the ground state is the protonation state of two titratable groups—the D85 counterion and the proton release group [66] located close to the extracellular side of the protein that includes E194, E204, R82, Y83, and surrounding water molecules, as shown in Figure 4c. In the O intermediate, the proton is located on the counterion, and it is transferred to the proton release group during the O → ground state transition. As suggested in a recent computational investigation [67], the proton transfer can proceed through the chain of water molecules shown in Figure 4c. While the direct experimental measurements of the counterion pKa in the O intermediate were not reported, a titration of bacteriorhodopsin showed that when the proton release group is deprotonated, the pKa of the counterion raises up to ≈7.5 [68]. Accordingly, the fluorescence quantum yield ($\approx 1\times 10^{-3}$) and the lifetime (9±2 ps) of the O intermediate at neutral pH are closer to the bacteriorhodopsin (pKa (D85) ≈ 2.7) at pH ≈ 2 (blue form) than at neutral pH.

Another fluorescent transient species, the Q intermediate, was detected by time-resolved fluorescence spectroscopy as a species with long-lived fluorescence (62±2 ps) [25,28,34]; its fluorescence quantum yield was measured to be $7\times 10^{-3}$, which is 7 times larger than the value for the O intermediate. The Q intermediate is not involved in the main photocycle of bacteriorhodopsin, but rather produced upon photoexcitation of the N intermediate [25]. The structure and protonation state of titratable residues of Q intermediate have not been determined, but the observed long-lived fluorescence allows us to suggest that the counterion in this form is protonated.

Long-lived fluorescence of photocycle intermediates is also detected for archaerhodopsin-3 [24,69], *N. pharaonius* halorhodopsin, Krokinobacter rhodopsin 2 (KR2), and rhodopsin from *Rubrobacter xylanophilus* (RxR) [24]. Under low intensity of a light source, only a dim fluorescence, which can be attributed to the ground state, is observed. However, under intense illumination favoring accumulation of photocycle intermediates, the fluorescence signal substantially increases. This intense fluorescence can be characterized by a bi-exponential decay with smaller and bigger components, $\tau_{1}$ and $\tau_{2}$ (Table 1). For the investigated rhodopsins, the $\tau_{1}$ and $\tau_{2}$ components range from 5.2 to 9.6 ps and from 24 to 60 ps, respectively. These values are similar to the lifetimes reported for the O and Q bacteriorhodopsin intermediates and can be attributed to the lifetimes for the O and Q states of the studied proteins.

For bright archaerhodopsin-3 mutants (Archer1, QuasAr1, and Archon2), which are nowadays widely used as genetically encoded voltage indicators, the origin of fluorescence enhancement is not completely understood yet. In QuasAr1 and Archon2, one of the counterions, D95, is substituted with glutamine and histidine, respectively, but the protonation state of the second counterion, D222, has not been investigated. Intense fluorescence of these proteins at physiological pH may also indicate the elevated pKa value of the remaining counterion and, consequently, its protonation. For Archer1, the increase in the counterion pKa up to 8.9 also has been recently demonstrated [42].

*Fluorescence emission and excitation bands maxima and Stokes shifts*. For majority of the discussed rhodopsins, the excitation band maximum $\lambda_{ex}$, i.e., an excitation wavelength at which the arising fluorescence is the most intense, is very similar to the absorption band maximum $\lambda_{abs}$ of the corresponding rhodopsin (Table 1). This observation is of particular importance for engineered bright rhodopsins QuasAr1 and Archer1, confirming that the fluorescence originates from the ground state but not from intermediates.

Two microbial rhodopsins, however, stand out from the rest. For *E. sibiricum* rhodopsin, the fluorescence excitation band is found to be red-shifted relative to the absorption band ($\lambda_{abs}$ = 531 nm, $\lambda_{exc}$ = 556 nm at pH = 7) [47]. To explain this finding, in the study [47], the excitation band was deconvoluted into two components. The red-shifted component (564 nm) was assigned to the brighter species with the protonated counterion D85 and the blue-shifted component (531 nm) was assigned to the dim species with the counterion in its deprotonated state. In line with this assumption, $\lambda_{em}$ is shifting closer to $\lambda_{abs}$ at a more alkaline pH ($\lambda_{exc}$ = 538 nm, $\lambda_{abs}$ = 531 nm, pH = 8.8). Moreover, for the ESR D85N mutant where the counterion is substituted with a polar residue, $\lambda_{exc}$ (564 nm) and $\lambda_{abs}$ (563 nm) are almost identical to the $\lambda_{exc}$ of the red-shifted component of the wild-type protein.

The observed shift of excitation and absorption bands for proteorhodopsin at pH = 7 ($\lambda_{abs}$ = 531 nm, $\lambda_{exc}$ = 564 nm) [43] can be also explained by coexisting states with the protonated and deprotonated counterion D97, which is in full agreement with its reported pKa value of ∼7.0 [72,73] and the multi-phasic decays of the excited-state population of this rhodopsin discussed in recent studies [30,63].

**Figure 5:**
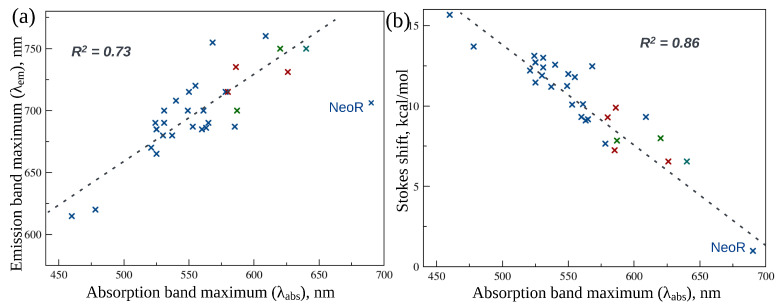
(**a**) Emission band maxima ($\lambda_{em}$) of microbial rhodopsins as a function of absorption band maxima ($\lambda_{abs}$). The data were fitted by the least squares method (the black dotted line); the point corresponding to NeoR was excluded from the data set. The color code is defined as in Figure 2. (**b**) Stokes shifts (in kcal/mol) of microbial rhodopsins as a function of $\lambda_{abs}$. The data were fitted by the least squares method (the black dotted line). The color code is defined as in Figure 2.

The emission band maxima of microbial rhodopsins demonstrate a general trend of increasing $\lambda_{em}$ upon red-shifting absorption band maxima (Figure 5a, but the linear correlation is moderate. The coefficient of determination $R^{2}$ is 0.73 if the data for NeoR were not included in the data set. However, for Stokes shifts (in kcal/mol) plotted as a function of $\lambda_{abs}$, the linear correlation is much better (Figure 5b). For this plot, the point corresponding to NeoR agrees perfectly with the rest of the microbial rhodopsins, and the coefficient of determination $R^{2}$ derived for the complete set is 0.86. Similar correlations were also observed for the data set that includes only the wild-type NeoR and its mutants [11].

The general trend of concurrent red-shifting for $\lambda_{abs}$ and $\lambda_{em}$ suggests that the difference in the charge distribution along the chromophore chain in $S_{0}$ and $S_{1}$ states, which is confirmed by analysis of the PSB electronic structures in the $S_{0}$ minimum and in the FC region reported in a number of computational studies, take place also in the $S_{1}$ minimum region of these surfaces (see Figure 1). The charge transfer character of the $S_{0}\to S_{1}$ transition was found to be responsible for the high sensitivity of $\lambda_{abs}$ to charged and polar residues in the protein environment (see [60] and references therein for a more detailed explanation). Similar sensitivity, although to a lesser extent, is observed for $\lambda_{em}$ suggesting the same mechanism for electrostatic tuning of the florescence band. However, more studies are required for detailed investigation of the role for both electrostatic and steric factors in the tuning of rhodopsin fluorescence properties.

## 3. Animal Opsin-Based Photoreceptors

Animal opsin-based photoreceptors perform a variety of roles related to sensation of light, such as vision, control of circadian rhythms, change in body color, etc. In the majority of cases, the light-activated functioning cycle of animal opsin-based photoreceptors involves the formation of the active state, which binds G-protein to launch signal transduction cascades [8,65]. Similarly to microbial rhodopsins, the central role in the photoactivation of animal opsin-based photoreceptors plays a chromophore that captures a photon and undergoes isomerization to start the corresponding photocycle. To date, four types of chromophores have been identified in animal opsin-based photoreceptors. In addition to retinal, 11-*cis* 3,4-dehydroretinal was found in visual opsin-based photoreceptors of fresh-water vertebrates, and 11-*cis* 3-hydroxy- and 4-hydroxyretinal were found in the photoreceptors of invertebrates.

*Monostable animal opsin-based photoreceptors*. In monostable animal opsin-based photoreceptors, the active state is thermally unstable and its deactivation involves release of the chromophore. Subsequently, the original inactive state is regenerated when opsin binds a chromophore from the medium [6,65,74]. The main representatives of monostable opsin-based photoreceptors are the visual rhodopsins of vertebrates. Available experimental results on fluorescent properties of monostable rhodopsins are scarce and available, to our knowledge, only for the bovine visual rhodopsin containing 11-*cis* retinal chromophore and its analogue with 9-*cis* retinal, isorhodopsin.

**Figure 6:**
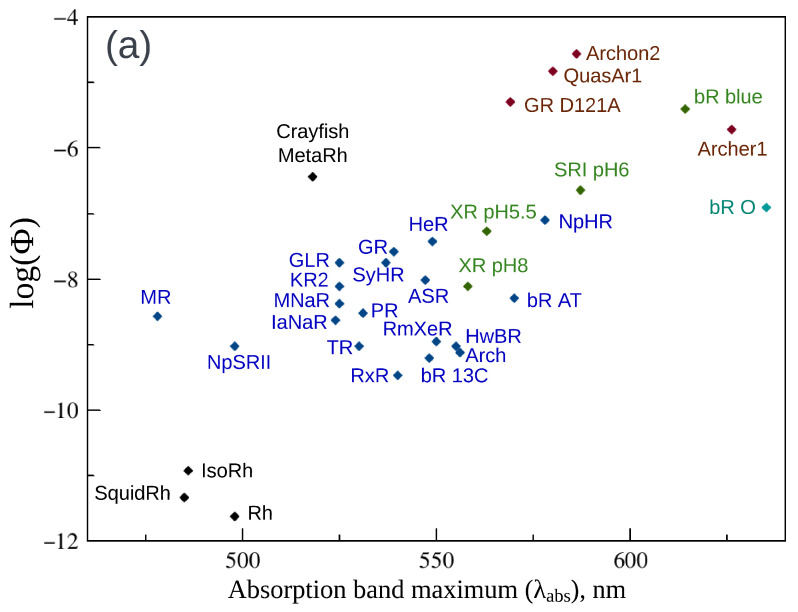

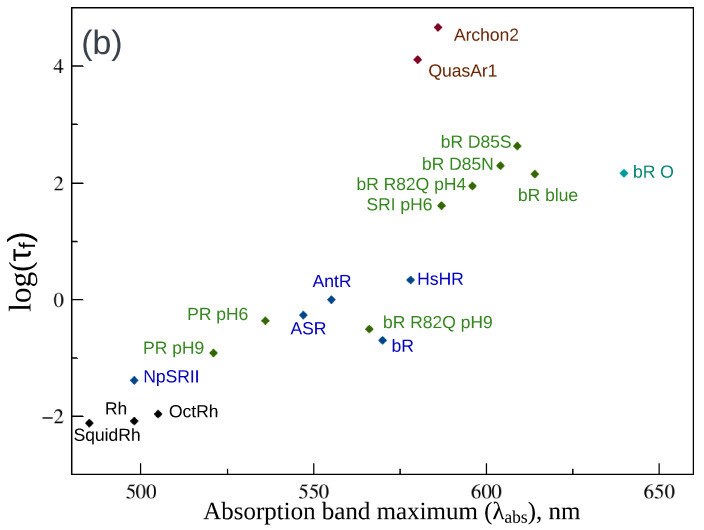
(**a**) Fluorescence quantum yields logarithm (log Φ) of microbial and animal rhodopsins as a function of absorption band maxima ($\lambda_{abs}$). (**b**) Fluorescence lifetimes logarithm (log $\tau_{f}$) of microbial and animal rhodopsins as a function of $\lambda_{abs}$. Animal rhodopsins and their photocycle intermediates are shown in black. The color code for microbial rhodopsins is defined as in Figure 2. For abbreviations of proteins, see Table 1 and Table 2.

The excited state decay of purified samples of bovine rhodopsin investigated with femtosecond up-conversion fluorescence spectroscopy was found to contain components with lifetimes of 0.146, 1.5, and 50 ps [75]. The 0.146 ps component with the largest contribution (80%) agrees with the fluorescence lifetime ≈0.1 ps, which was evaluated using the fluorescence quantum yield value ($\Phi =1.3\pm 0.4\times 10^{-5}$) measured for rod outer segments by a comparative method with erythrosin as a reference [76]. In the same up-conversion experiment, the repeated excitation of purified rhodopsin samples resulted in a linear dependence of the fluorescence intensity on the excitation power that indicates that photocycle intermediates do not contribute into fluorescent signal [75]. This observation is in line with results obtained in an earlier study [77], which detected the fluorescence of bovine rhodopsin photocycle intermediates, but evaluated it to be much lower than fluorescence of the ground state form of rhodopsin photocycle. The fluorescence quantum yield of isorhodopsin was found to be only 2-fold larger compared to that of rhodopsin [78]. Thus, the fluorescence detected in the bovine rhodopsin and isorhodopsin is even less intense than the fluorescence of the wild-type microbial rhodopsins and the corresponding black points are located in the bottom left corner in Figure 6a,b.

*Bistable animal opsin-based photoreceptors*. Just as in monostable animal opsin-based photoreceptors, bistable animal opsins bind a retinal chromophore that captures light to initiate G protein-mediated phototransduction cascades. However, for this type of opsin-based photoactive proteins, a release of the retinal chromophore and bleaching do not occur during the photocycle but another thermally stable photoproduct, metarhodopsin, forms instead. Absorption of a second photon can convert metarhodopsin back to the ground state form. Irradiation with light of different wavelengths allows controlling the ratio of these two forms in the photostationary state. Bistable animal opsin-based photoactive proteins are widely spread in the animal world and serve not only as visual receptors of invertebrates but also perform many other functions [65,79,80].

As is the case with vertebrate visual pigments, the available data on fluorescence properties of animal bistable opsins are much more scarce than for microbial rhodopsins (Table 2). So far, to our knowledge, the fluorescence quantum yields are measured only for the ground state of squid visual rhodopsin [76] and for the meta-form of visual rhodopsin in crayfish [81]; fluorescence lifetimes are reported for the ground states of squid [76] and octopus rhodopsins [82]. All of the aforementioned opsins bind 11-*cis* retinal in their ground state that photoisomerizes to the all-*trans* isomer in corresponding metarhodopsins. The quantum yield measured for the ground state of squid rhodopsin that was extracted from the eyes of this invertebrate species was found to be $\Phi =1.2\times 10^{-5}$ [76] (a black point in the bottom left corner of Figure 6a and Table 2). This number is lower than the fluorescence quantum yields of bacteriorhodopsin and comparable with the quantum yields of bovine rhodopsin and isorhodopsin. On the contrary, the fluorescence quantum yield of the crayfish metarhodopsin recorded by microspectrofluorometry from isolated photoreceptor organelles (rhabdoms) at neutral pH 7.5 is considerably higher ($\Phi =1.6\pm 0.4\times 10^{-3}$ [81]) and the corresponding black point in Figure 6a lies far above the points that represent ground states of both natural microbial and visual rhodopsins at physiological pH and closer to the points that correspond to the bacteriorhodopsin O intermediate, the acidic form of bacteriorhodopsin, and the engineered fluorescent mutants of microbial rhodopsins. Although the fluorescence quantum yield for the ground state of the crayfish visual rhodopsin was not reported either in the discussed study [81] or in more recent studies; the authors also performed a thorough investigation to conclude that the fluorescence of the dark-adapted sample, i.e., the ground state of crayfish rhodopsin, was much lower than the fluorescence of the light-adapted sample and could not be detected with techniques applied in this study.

**Table 2:** Fluorescence properties of animal rhodopsins.

Protein	$\lambda_{\mathrm{abs}}$, nm	$\lambda_{\mathrm{exc}}$, nm	$\lambda_{\mathrm{em}}$, nm	Stokes Shift, nm	Φ	$\tau_{f}$, ps
Rh	498 [76]	-	600 [76]	102	$1.2\times 10^{-5}$ [76] $0.9\times 10^{-5}$ [78]	0.05 [78] 0.146, 1.5 [75] 0.125, 1 [83]
IsoRh	485 [78]	-	≈620 [78]	135	$1.8\times 10^{-5}$ [78]	-
SquidRh	485 [76]	-	620 [76]	135	$1.2\times 10^{-5}$ [76]	0.12 [76]
OctRh	505 [82]	-	-	-	-	0.14 [82]
Batho	543 [84]	-	-	-	<10${}^{-5}$ [84]	-
Lumi	497 [77]	-	600	103	<10${}^{-5}$ [77]	-
MetaI	478 [77]	-	580	102	<10${}^{-5}$ [77]	-
MetaII	380 [77]	-	500–515	120	<10${}^{-5}$ [77]	-
Crayfish meta	-	518 [81]	660–670 [81]	142	$1.6\times 10^{-3}$ [81]	-
Blowfly meta	584 (M) 568 (M${}^{\prime }$) [85]	-	660 [85]	92	Φ(M${}^{\prime }$)> 3Φ(M) [85]	-
Housefly meta	580 (M) 570 (M${}^{\prime }$) [86]	-	660 [86]	90	-	-
Droso meta	-	570 [87]	>646 [87]	-	-	-

$\lambda_{abs}$—absorption band maximum, $\lambda_{exc}$—fluorescence excitation band maximum, $\lambda_{em}$—emission band maximum, Φ—fluorescence quantum yield, $\tau_{f}$—fluorescence lifetime. Abbreviations: Rh, bovine (*Bos Taurus*) rhodopsin; IsoRh, bovine isorhodopsin; SquidRh, squid (*Todarodes Pacificus*) rhodopsin; OctRh, octopus (*Paroctopus Defleini*) rhodopsin; Batho, bovine bathorhodopsin; Lumi, bovine lumirhodopsin; MetaI and MetaII, bovine metarhodopsins I and II; Crayfish meta, metarhodopsin from crayfish *Orconectes rusticus*; Blowfly meta, metaxanthopsin from blowfly *Calliphora erythrocephala*; Housefly meta, metaxanthopsin from housefly *Musca domestica*; Droso meta, metaxanthopsin from *Drosophila melanogaster*.

A similar substantial increase in fluorescence of meta-states comparing to the ground state was observed also in in vivo microspectrofluorometric studies on the housefly [86] (*Musca domestica*), Drosophila [87] (*Drosophila melanogaster*), and blowfly [85] (*Calliphora erythrocephala*) visual pigments. For housefly and blowfly, two distinct meta-states, M and M${}^{\prime }$, were detected [85,86]. For housefly, the relative fluorescence quantum yield of M and M’ forms was also measured and the ratio Φ(M)/$\Phi (M^{\prime })$ was found to be >3. The authors suggested that M and M’ meta-forms differ by *trans*- and *cis*- isomers of the 3-hydroxy-retinal, which serves as the chromophore in this species.

For bistable pigments, excited state lifetimes were measured for the octopus rhodopsin (*Paroctopus Delheini*) [82] and for the squid rhodopsin [76] and were found to be 0.14±0.07 ps and 0.12±0.05 ps, respectively. As for the bovine rhodopsin, these values are lower than the lifetimes reported for microbial rhodopsins and the corresponding points are located in the bottom of Figure 6b.

In addition, it is worth paying some attention to studies that deal with the photochemical properties of the protonated Schiff base other than fluorescence but that can be related to fluorescence efficiency. In a comprehensive spectroscopic study on photochemical properties of the mouse melanopsin [88], a bistable non-visual animal opsin with 11-*cis* retinal in its most thermodynamically stable ground state ($\lambda_{abs}=467$ nm) and all-*trans* retinal in the meta-state ($\lambda_{abs}=476$ nm), the photoisomerization quantum yield of ground-state melanopsin was found to be more than twice larger than for metamelanopsin: 0.52±0.02 and 0.22±0.01, respectively. The photoisomerization quantum yield reported for the bovine visual rhodopsin (0.65±0.01 [89]) is closer to the photoisomerization quantum yield measured for the ground form of the mouse melanopsin than for the meta-form of this melanopsin.

Recent studies [90,91] on photochemical properties of Rhodopsin-1 from the jumping spider *Hasarius adansoni* (JSR1), which possesses 11-*cis* retinal in its ground state ($\lambda_{abs}=535$ nm) and all-*trans* retinal in meta-state (Meta-JSR1) with a similar absorption band maximum ($\lambda_{abs}=535$) nm but slightly bigger extinction coefficient [91], do not contain any information about fluorescence properties of either JSR1 or Meta-JSR1. However, simulations of the photostationary state performed in Ref. [90] suggested lower quantum efficiencies for the reverse Meta-JSR1 → JSR1 transitions (0.4–0.5) than for the direct JSR1 → Meta-JSR1 transitions (0.7). These findings are again in line with the assumption that the photoisomerization process occurs more efficiently if a negative charge is present in the vicinity of ${}^{+}$N-H part of the retinal protonated Schiff base.

**Figure 7:**
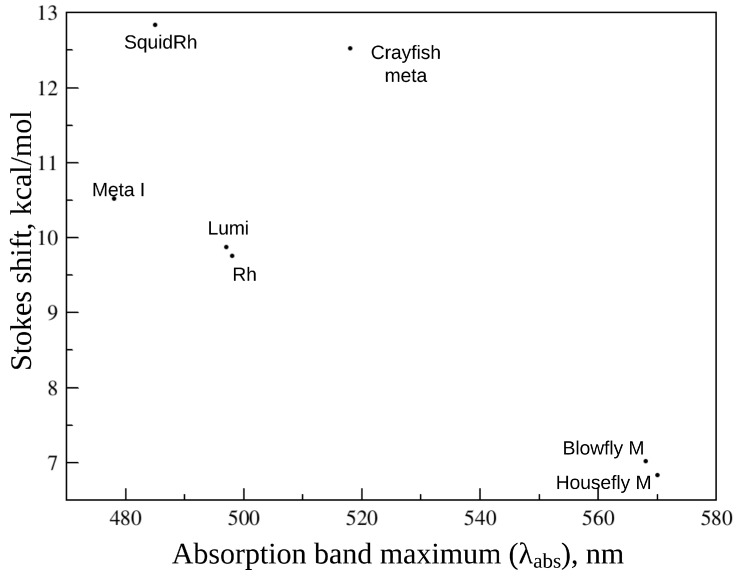
Stokes shifts (in kcal/mol) of animal rhodopsins and their photocycle intermediates as a function of $\lambda_{abs}$.

*Fluorescence emission and excitation band maxima and the Stokes shifts*. As is the case for microbial rhodopsins, the general trend of decreasing Stokes shifts at longer absorption bands maxima wavelengths is observed for animal opsin-based photosensitive proteins. However, the available data are not sufficient to unambiguously derive any correlation (Figure 7).

## 4. Retinal Protonated Schiff Based in the Gas Phase and Solvents

Recently, the excited state decay of the retinal protonated Schiff base (PSB) has been studied in the gas phase [92]. Due to extreme instability of the PSB in the gas phase, a special experimental approach combining time-resolved action spectroscopy with femtosecond pump-probe techniques in an ion-storage ring has been applied. The observed excited state decay was fitted by two components with ≈0.4 ps and 3 ps lifetimes at room temperature and ≈1.4 ps and 77 ps at 100 K. The fast and slow components were assigned to *cis* (most likely 11-*cis*) and all-*trans* forms of the chromophore, respectively. The temperature dependence was explained by potential energy barriers on the excited state potential energy surface that has to be overcome to achieve the conical intersection. This assumption was also supported by ab initio calculations.

**Figure 8:**
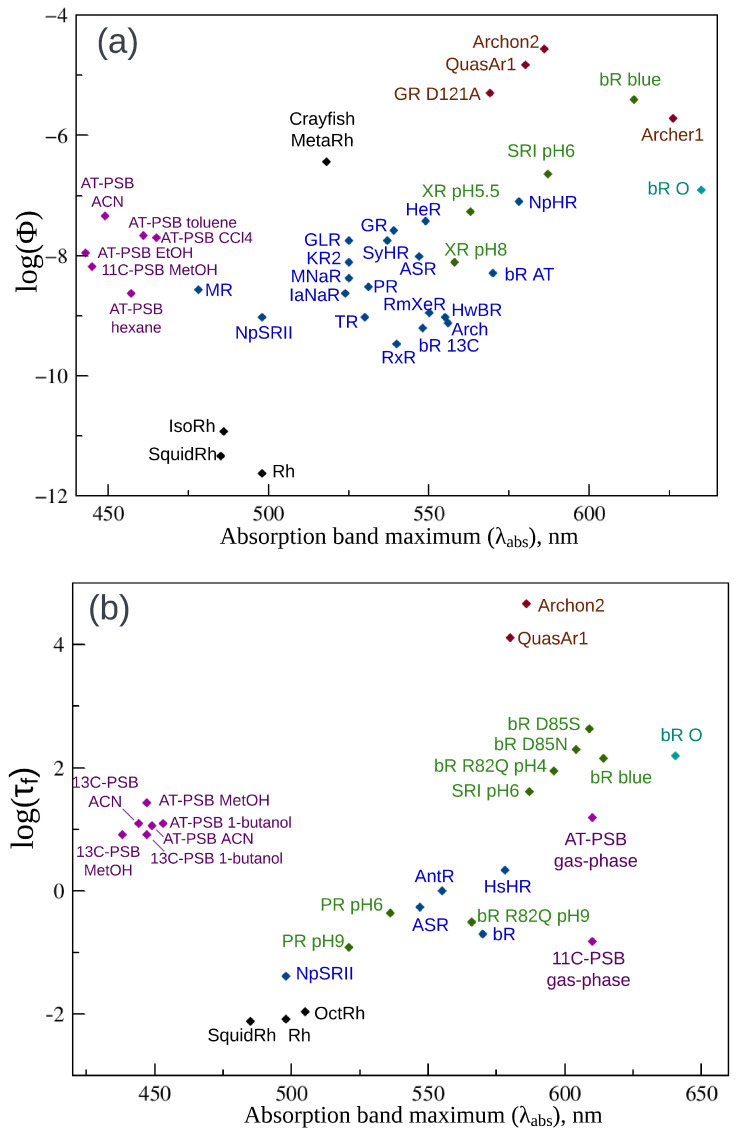
(**a**) Fluorescence quantum yields logarithm (log Φ) of microbial, animal rhodopsins, and retinal protonated Schiff base (PSB) isomers in solvents and gas phase as a function of absorption band maxima ($\lambda_{abs}$). (**b**) Fluorescence lifetimes logarithm (log $\tau_{f}$) of microbial, animal rhodopsins and retinal PSB isomers in solvents and gas phase as a function of $\lambda_{abs}$. PSB isomers in solvents and gas phase are shown in purple. The color code for rhodopsins is defined as in Figure 6. For abbreviations of proteins, see Table 1 and Table 2.

The discussed combined experimental and computational study on PSB in the gas phase is of particular interest to rhodopsin photochemistry research since it provides information about the intrinsic photochemical properties of the chromophore in the absence of any interactions with protein or solvent environments. In Figure 8b, the points that represent the all-*trans* and 11-*cis* retinal PSB isomers in the gas phase are added to the previously discussed lifetimes of microbial and animal rhodopsins. Expectedly, the point corresponding to the all-*trans* isomer is located closer to the blue form of bacteriorhodopsin with the neutralized counterion than to the points of microbial rhodopsins at neutral pH; the point corresponding to the 11-*cis* chromophore also lies above the points corresponding to animal rhodopsins with a deprotonated counterion but lower than the point of the all-*trans* isomer.

Finally, we considered studies on the photoisomerization properties of the PSB (more precisely, of the PSB-counterion complexes) in different solvents. The data are compiled in Table 3 and Figure 8a,b. The points corresponding to the retinal PSB in different solvents are located at the left sides of Figure 8a,b since all chromophore isomers possess significantly blue-shifted absorption bands in solvents. Two patterns can be seen in Figure 8. Just as for rhodopsins and gas-phase PSBs, if data for the same solvent considered, the points corresponding to the 11-*cis* or 13-*cis* isomers lie below the points corresponding to the all-*trans* isomer. The fluorescence quantum yield of the all-*trans* isomer measured in a non-polar and low-polarizable solvent, hexane (Figure 8a), is lower than the quantum yield measured in polar or polarizable solvents. Apparently, in hexane shielding of the counterion may be lower than in polar and polarizable solvents but additional computational studies are required to test this assumption.

## 5. Neorhodopsin

Optical and photophysical properties of the recently discovered Neorhodopsin, which is one of two subunits of rhodopsin-cyclases from Chytridiomycota fungi, stands outs from the rest of the microbial rhodopsins. Although this protein possesses the same all-*trans* chromophore as the rest of the microbal rhodopsins, the absorption spectral band is not only shifted to the near-infrared region but also demonstrates complex shape, which more resembles the absorption band of the green fluorescent protein than absorption bands of the rest of the microbial rhodopsins. The most prominent feature of NeoR is its very high fluorescence quantum yield (20%). To date, the fluorescence properties of NeoR are well characterized experimentally and the data for a number of its mutants are also available. Still, more studies to evaluate the structural reasons for its unique spectral and photophysical properties are required. The fluorescence properties of this protein along with other characteristics have been reviewed very recently in Ref. [11].

## 6. Conclusions

Fluorescence and photoisomerization of the chromophore in opsin-based photosensitive proteins have been investigated for decades [7,8,98,99]. Recently, the research in this area has been facilitated by application of rhodopsins as tools for optogenetics [100,101,102], which is, along with photopharmacology [103,104,105,106], a widely used approach to control and monitor biological cell activity using light. Although high fluorescence and, accordingly, inefficient photoisomerization of the chromophore in rhodopsins is an impediment for application of these proteins as actuators, intense fluorescence is a desirable feature for their applications as genetically encoded voltage indicators. For the rational engineering of new variants of bright fluorescent rhodopsins, a detailed understanding of how the protein environment tunes fluorescence properties is required. Moreover, the knowledge of how a modification of protein composition or structure affects different fluorescence properties can be used to gain a structural insight into rhodopsins and their photocycle intermediates based on fluorescence measurements.

The goal of this review is the compilation and analysis of available experimental data to identify and highlight main points that can be considered general for a variety of microbial and animal opsin-based photosensitive proteins. The vast majority of the available studies are performed for rhodopsins, i.e., opsins in which the retinal Schiff base serves as the cofactor. A few studies on visual pigments of the housefly, blowfly, and Drosophila with the 3-hydroxy-retinal chromophore are also available and the data coincide well with the data for rhodopsins.

The all-*trans*-form of the chromophores of opsin-based photoactive proteins demonstrate slower excited state decay than the 11-*cis*-form in the gas phase. A similar trend is preserved for rhodopsins and the retinal PSB in solvents: the fluorescence lifetime and fluorescence quantum yield is higher for rhodopsins with the all-*trans*-form of the retinal than for 11-*cis* or 13-*cis*-forms of the retinal PSB, assuming that the electrostatic environment is similar. A drastic change in the fluorescence quantum yield and fluorescence lifetime is observed after transition to rhodopsins with a neutralized counterion, i.e., to acidic forms, the O photocycle intermediate of microbial rhodopsins or meta-states of animal rhodopsins. Generally, one can conclude that the decrease in the stabilization of a positive charge in the ${}^{+}$N-H part of the chromophore leads to enhanced fluorescence and slower excited state decay. In this context, the role of the counterion electrostatic effect is an important factor to achieve fluorescence enhancement. Analysis of the dependence of fluorescence efficiency on absorption band maxima reveals a clear trend of fluorescence enhancement with red-shifting of absorption bands but not a strong correlation. Surprisingly, a moderately strong linear correlation ($R^{2}$= 0.86) exists between the Stokes shifts of microbial rhodopsins and absorption band maxima. Obviously, both the electrostatic and steric interactions of the chromophore with the protein environment can tune fluorescence properties. Although a counterion contribution to the electrostatic field in the chromophore region is decisive, the electrostatic effect of other charged and polar residues can also be significant. The steric interaction of the chromophore with surrounding residues of the binding pocket also can be an important factor for tuning the fluorescence properties. Further theoretical and experimental studies are needed to advance this subject.

## Figures and Tables

**Table 1:** Fluorescence properties of microbial rhodopsins.

Protein	$\lambda_{\mathrm{abs}}$, nm	$\lambda_{\mathrm{exc}}$, nm	$\lambda_{\mathrm{em}}$, nm	Stokes Shift, nm	Φ	$\tau_{f}$, ps
bR	568 [25,26]	570 [27]	755 ± 10 [28]	187 ± 10	2.5–2.7 × 10${}^{-4}$ (AT) 0.7–1.2 × 10${}^{-4}$ (13C) [29]	0.5 [26] 0.56 [30]
bR blue	610–620 [31]	613–614 [31]	750 [29]	130-140	$4.5\times 10^{-3}$ [31]	1.5, 8.6 [32] 7.8 [30]
bR O	635–640 [33]	-	750±5 [28]	110–120	≈1 $\times 10^{-3}$ [34]	9±2 [34]
bR Q	-	-	<720 [28]	-	Φ≈110×Φ(bR) [25] $7\times 10^{-3}$ [34]	62±2 [34]
bR D85S	609 [35]	-	760 [35]	161	-	3.6, 14 [35]
bR D85N	604 [36]	-	-	-	-	2.0, 10 [36]
bR R82Q, pH4.4	596 [36]	-	-	-	-	2.0, 7 [36]
bR R82Q, pH9.6	556 [36]	-	-	-	-	0.6 [36]
Arch	553 [24]	553 [24]	687 [24]	134	$1.1\times 10^{-4}$ [24] $9\times 10^{-4}$ [37]	5.9, 60 (intense illumination) [24]
Arch D95N	585 [37]	-	687 [37]	102	$4\times 10^{-4}$ [37]	-
QuasAr1	580 [23,38]	590 [39] 585 [38]	715 [39] 740 [38]	135, 160	$8\times 10^{-3}$ [39] $6.5\times 10^{-3}$ [38]	61.5 [38]
Archon2	586 [40]	-	735 [40]	149	$1.05\times 10^{-2}$ [40]	106 [40]
Archer1	626 [41]	627 [42]	731 [41]	103	$3.3\times 10^{-3}$ [41]	-
PR	531 [13]	565 [13]	700 [13]	169	$2\times 10^{-4}$ [13]	1.4 [13] 0.7, 15 (pH6) 0.4, 8 (pH9) [43]
XR	560 [44]	-	685 [44]	125	$3\times 10^{-4}$ (pH8) $7\times 10^{-4}$ (pH5.5) [44]	-
GR	541 [45]	568 (pH4.5) [45] 550 [24]	670 (pH7.2) 680 (pH4.5) [45] 723 [24]	129 (pH7.2) 135 (pH4.5) 173 [24]	F(pH4.5)= 4F(pH7.2) [45] $5.1\times 10^{-4}$ [24]	9.6, 47 (intense illumination) [24]
GR D121A	≈568 [46]	-	-	-	$5\times 10^{-3}$ [46]	-
ESR	546 (pH2) 534 (pH5) 531 (pH7) 521 (pH10.5) [47]	565 (pH5) 556 (pH7) 538 (pH8.8) [47]	≈690 [47]	≈159	F(pH5) = 2F(pH7) [47]	-
ESR D85N	563 (pH5) [47]	564 (pH5) [47]	686 [47]	123	F=7F(ESR) [47]	-
ESR H57M	565 (pH5) 517 (pH8.5) [47]	568 (pH4.5) 520 (pH8.8) [47]	690 (pH4.5) 650 (pH7.5) [47]	125 (pH4.5) 133 (pH8)	F(pH4.5)≈ 100F(pH9) [47]	-
HeR	549 [48]	-	≈700 [48]	151	$6\times 10^{-4}$ [48]	-
ASR	541 (pH7) [24] 547 (AT) [49] 533 (13C) [49]	540 (pH7) [24]	700 [24]	160	$3.3\times 10^{-4}$ (AT) $0.8\times 10^{-4}$ (13C) [50]	0.1, 0.77 [51]
KR2	525 [24]	525 [24]	685 [24]	160	$3\times 10^{-4}$ [24]	8.2, 46 (intense illumination) [24]
RxR	540 [24]	540 [24]	708 [24]	168	$0.77\times 10^{-4}$ [24]	5.2, 54 (intense illumination) [24]
NpHR	576 [24]	576 [24]	715 [24,52] ≈750 [53]	115 150	$8.3\times 10^{-4}$ [24] $5\times 10^{-4}$ [53]	6.7, 24 (intense illumination) [24] 0.17, 1.5, 8.5 [54] 2.3 [52]
GLR	525 [24]	520 [24]	685 [24]	165	$4.3\times 10^{-4}$ [24]	-
HwBR	555 [24]	550 [24]	720 [24]	170 [24]	$1.2\times 10^{-4}$ [24]	-
IaNaR	524 [24]	525 [24]	690 [24]	165 [24]	$1.8\times 10^{-4}$ [24]	-
MNaR	525 [24]	500 [24]	665 [24]	165 [24]	$2.3\times 10^{-4}$ [24]	-
MR	478 [24]	460 [24]	620 [24]	160 [24]	$1.9\times 10^{-4}$ [24]	-
HsHR	578 [54]	-	-	-	-	1.5. 8.5 [54]
NpSRII	498 [24,52] 460 [52]	460 [24]	615 [24] 630 [52]	155 [24] 132 [52]	$1.2\times 10^{-4}$ [24,55]	0.25, 3.0 [52]
SRI pH6	587 [55]	-	700 [55]	113	≈1.3 $\times 10^{-3}$ [55]	5, 33 [55]
RmXeR	550 [24]	545 [24]	715 [24]	170 [24]	$1.3\times 10^{-4}$ [24]	-
SyHR	537 [24]	535 [24]	680 [24]	145 [24]	$4.3\times 10^{-4}$ [24]	-
TR	530 [24]	518 [24]	680 [24]	162 [24]	$1.2\times 10^{-4}$ [24]	-
AntR	555 [56]	-	-	-	-	1, 5 [56]
NeoR	690 [11]	-	707 [11]	17	0.2 [11]	1100 [11]

$\lambda_{abs}$—absorption band maximum, $\lambda_{exc}$—fluorescence excitation band maximum, $\lambda_{em}$—emission band maximum, Φ—fluorescence quantum yield, $\tau_{f}$—fluorescence lifetime. For biexponential decays, both $\tau_{f}$ values are given separated by a comma. Abbreviations: bR, *Halobacterium halobium* bacteriorhodopsin; AT, *all-trans* form; 13C, *13-cis* form; bR blue, blue form of bR at pH2.6; bR O and bR Q, O and Q intermediates of bR photocycle; Arch, archaerhodopsin-3; QuasAr1, Archon2, Archer1, bright mutants of archaerhodopsin-3; PR, proteorhodopsin from marine γ-proteobacteria; XR, xanthorhodopsin; GR, *Gloebacter violaceus* rhodopsin; ESR, *Exiguobacterium sibiricum* rhodopsin; HeR, heliorhodopsin. (int)—fluorescence lifetimes detected under intense illumination. Abbreviations: ASR, Anabaena sensory rhodopsin; KR2, Krokinobacter rhodopsin 2 from *Krokinobacter eikastus*; RxR, rhodopsin from *Rubrobacter xylanophilus*; NpHR, halorhodopsin from *Natronomonas pharaonis*; GLR, sodium pumping rhodopsin from *Gillisia limnaea*; HwBR, bacteriorhodopsin from *Haloquadratum walsbyi*; IaNaR, sodium pumping rhodopsin from *Indibacter alkaliphilus*; MNaR, sodium pumping rhodopsin from *Micromonospora* sp. CNB394; MR, middle rhodopsin from *Haloquadratum walsbyi*; HsHR, halorhodopsin from *Halobacterium salinarum*; SRII, sensory rhodopsin II from *Natronobacterium pharaonis*; SRI, sensory rhoodopsin I from *Halobacterium salinarum*; RmXer, xenorhodopsin from *Rubricoccus marinus*; SyHR, Synechocystis halorhodopsin from *Synechocystis* sp. PCC 7509; TR, thermophilic rhodopsin from *Thermus thermophilus* JL-18; AntR, antarctic rhodopsin.

**Table 3:** Fluorescence properties of retinal in the gas-phase and solvents.

Species	Solvent	$\lambda_{\mathrm{abs}}$, nm	$\lambda_{\mathrm{em}}$, nm	Stokes Shift, nm	Φ	$\tau_{f}$
11C-PSB	gas-phase	610 [92]	-	-	-	0.442 ± 0.121 ps [92,93]
AT-PSB	gas-phase	610 [92]	-	-	-	3.3 ± 1 ps [92]
AT-PSB	methanol	445 [94] 442 [95] 447 [96]	655 [94] 675 [95] 630 [96]	210 [94] 233 [95] 183 [96]	-	90 fs (525) 0.5, 2.8 ps (605) 3.7 ps (762) [94] <0.2, 4.2 ps [96]
13C-PSB	methanol	438 [96]	630 [96]	192	-	2.5 ps
11C-PSB	methanol	445 [97]	660 [97]	215	$2.8\times 10^{-4}$ [97]	0.5, 2.0 ps (605) 3.1 ps (695) [97]
AT-PSB	acetonitrile	449 [96]	690 [95] 650 [96]	241 201	$6.5\times 10^{-4}$ [95]	<5 ps [95] 2.9 ps [96]
13C-PSB	acetonitrile	444 [96]	652 [96]	208	-	3.0 ps [96]
AT-PSB	1-butanol	453 [96]	610 [96]	157	-	3.0 ps [96]
13C-PSB	1-butanol	447 [96]	603 [96]	156	-	2.5 ps [96]
AT-PSB	hexane	457 [95]	620 [95]	163	$1.8\times 10^{-4}$ [95]	<5 ps [95]
AT-PSB	carbon tetrachloride	465 [95]	650 [95]	185	$4.5\times 10^{-4}$ [95]	-
AT-PSB	toluene	461 [95]	660 [95]	199	$4.7\times 10^{-4}$ [95]	-
AT-PSB	ethyl acetate	432 [95]	650 [95]	218	-	-
AT-PSB	acetone	447 [95]	680 [95]	233	-	-
AT-PSB	ethanol	443 [95]	660 [95]	217	$3.5\times 10^{-4}$ [95]	<6ps [95]
AT-PSB	propanol	449 [95]	650 [95]	201	-	-

$\lambda_{abs}$—absorption band maximum, $\lambda_{exc}$—fluorescence excitation band maximum, $\lambda_{em}$—emission band maximum, Φ—fluorescence quantum yield, $\tau_{f}$—fluorescence lifetime. Abbreviations: AT-PSB, 13C, and 11C-PSB—all-*trans*, 13-*cis*, and 11-*cis* retinal protonated Schiff base. Numbers in parenthesis denote illumination wavelength.

## Data Availability

Not applicable.
